# The role of bone marrow biopsy in patients with plasma cell disorders: should all patients with a monoclonal protein be biopsied?

**DOI:** 10.1038/s41408-020-0319-0

**Published:** 2020-05-06

**Authors:** M. Hasib Sidiqi, Mohammed Aljama, Shaji K. Kumar, Dragan Jevremovic, Francis K. Buadi, Rahma Warsame, Martha Q. Lacy, David Dingli, Wilson I. Gonsalves, Amie L. Fonder, Miriam A. Hobbs, Yi Lisa Hwa, Prashant Kapoor, Taxiarchis Kourelis, Nelson Leung, Eli Muchtar, John A. Lust, Robert A. Kyle, Ronald S. Go, Vincent S. Rajkumar, Morie A. Gertz, Angela Dispenzieri

**Affiliations:** 1 Division of Hematology, Department of Internal Medicine, Mayo Clinic, Rochester, MN USA; 2 Department of Hematology, Fiona Stanley Hospital, Perth, WA Australia; 3 Juravinski Cancer Centre, McMaster University, Hamilton, ON Canada; 4 Department of Laboratory Medicine and Pathology, Mayo Clinic, Rochester, MN USA; 5 Division of Nephrology and Hypertension, Mayo Clinic, Rochester, MN USA

**Keywords:** Myeloma, Myeloma

## Abstract

We conducted a retrospective review of multiple myeloma (MM), smoldering multiple myeloma (SMM), and monoclonal gammopathy of undetermined significance (MGUS) patients seen at Mayo Clinic to determine whether a bone marrow biopsy (BM) is necessary in all patients diagnosed with a monoclonal protein. A total of 2254 MM, 397 SMM, and 5836 MGUS patients were included in the study. A total of 29 (1.3%) MM patients “without CRAB/FLC” were identified where BM or advanced imaging was critical for diagnosis, 8 (0.3% MM cohort) of whom were diagnosed with MM solely on BM findings (plasma cells > 60%). Without BM or advanced imaging none of these patients would be classified low-risk MGUS. A total of 314 (79%) MGUS-like SMM patients were identified where classification of SMM was based on BM findings. Without BM 97 would be classified as low/low-intermediate-risk MGUS and 151 intermediate or high-risk MGUS; 66 had missing information precluding classification. Only three (<1% SMM cohort) were low-risk MGUS without abnormalities in hemoglobin, calcium, and renal function. In patients presenting with low-risk MGUS and normal hemoglobin, calcium, and renal function, the risk of missing a diagnosis of SMM and MM by omitting BM is <1%. BM should be deferred in these patients in preference to clinical and laboratory monitoring.

## Introduction

Multiple myeloma (MM) is a hematological malignancy that results from neoplastic proliferation of plasma cells in the bone marrow (BM). In 2018, ~30,770 people will be diagnosed with MM in the United States, accounting for 1.8% of all new cancer cases, and ~12,770 people will die from MM^1^. MM evolves from the premalignant state of monoclonal gammopathy of undetermined significance (MGUS)^2^. Progression from MGUS to MM may include an intermediate stage of disease, smoldering multiple myeloma (SMM), a heterogeneous entity comprising of a group of patients that behave biologically like MGUS and another group that eventually develop clinical manifestations of myeloma requiring therapy such as hypercalcemia, renal dysfunction, anemia and bone lesions (CRAB)^3^. Like MM, rates of MGUS prevalence increase with age: more than 4% in Caucasians over age 50 and greater than 6% in Caucasians over 70 years of age^4^. Prevalence rate for MGUS among African Americans is threefold higher than Caucasians. Patients with MGUS, SMM, and MM require comprehensive clinical and laboratory assessment to differentiate one from the other. This distinction is important because the first two diagnoses do not require active therapy, whereas the third does.

By definition, patients with MGUS or SMM are “asymptomatic,” but in reality many have symptoms that prompted the testing, but these symptoms are eventually deemed not to be related to their plasma cell disorder. Others arrive at a diagnosis due to an incidental finding of an elevated sedimentation rate or total protein. BM aspiration and biopsy (BM) and advanced imaging can help differentiate between entities^5^ and provide prognostic information^6–9^, but the frequency in which these expensive and/or invasive tests contribute changes in management has not been well delineated. Herein we report on patients with SMM and MM that present without symptoms and in whom BM was critical for diagnosis.

## Methods

We retrospectively reviewed all patients with SMM (*n* = 532) and MM (*n* = 2674) seen at the Mayo Clinic in Rochester between 1 January 2000 and 31 December 2016. To be eligible for this retrospective study, patients were required to have: (1) been diagnosed with SMM or MM between 1 January 2000 and 31 December 2016; (2) been seen at the Mayo Clinic within 90 days of diagnosis; and (3) have available data to assess whether any of the following CRAB features (CRAB) were present at diagnosis. Five thousand eight hundred and sixty-three patients with MGUS, who met above criteria, were also included as a comparator group. Patients with solitary plasmacytoma with or without minimal BM involvement were excluded. Patients were classified as SMM or MM according to the updated IMWG criteria^5^. Patients diagnosed prior to publication of these criteria were reclassified accordingly. The percent BM plasmacytosis was based on the highest value from the smear, aspirate, or BM biopsy^10^.

The Mayo MGUS risk score, the International Staging System for MM and the updated Mayo Clinic risk stratification model for SMM were utilized^6,11,12^. In brief, the MGUS risk score stratifies patients into four groups based on an abnormal kappa-to-lambda serum free light chain ratio, serum M-spike of ≥1.5 g/dL and non-IgM monoclonal isotype (low risk with no factors, low-intermediate risk with one factor, intermediate risk with two factors, and high risk with all three factors present). The SMM risk model stratifies patients into three groups based on an M-spike of >2 g/dL, involved-to-uninvolved serum-free light chain ratio of >20 and BM plasma cells >20% (low risk with none of the three risk factors, intermediate risk with one of the three risk factors, and high risk ≥2 of the three risk factors).

To better categorize the critical role of the BM in establishing the diagnosis we categorized patients with MM into two groups based on testing parameters for CRAB and the presence of serum immunoglobulin free light chain ratio (FLCr) of ≥100 and involved FLC level ≥ 100). MM patients classified as “without CRAB/FLC” had laboratory values within reference range for calcium, creatinine, hemoglobin, and absence of lytic lesions assessed by conventional skeletal survey and FLCr < 100. This is contrasting to the IMWG diagnostic criteria for CRAB features (anemia > 2 g/dL below normal or <10 g/dL, hypercalcemia > 1 mg/dL higher than normal or >11 mg/dL, and renal insufficiency with creatinine clearance <40 mL/min or creatinine of >2 mg/dL), since abnormalities outside of these stringent diagnostic criteria are likely to serve as triggers for further investigation in a patient with a monoclonal gammopathy. Those “with CRAB/FLC” had an abnormality detected in at least one of these variables. In patients without CRAB, chart rereview was conducted to further clarify the criteria used to establish the diagnosis of MM.

Patients with SMM were categorized based on serum M-protein and urine M-protein levels. Those SMM patients with a serum M-protein of ≥3 g/dL or urinary M-protein ≥ 500 mg per 24 h were classified as “MM-like” with the remaining patients classified as “MGUS-like.” Patients without data on the serum M-protein at time of diagnosis of SMM were excluded from this categorization.

Descriptive statistics with median, interquartile range (IQR), and range were used for continuous variables and proportions (%) for categorical variables. The Pearson χ^2^ and Fisher’s exact test were used to compare categorical variables and the Wilcoxon rank-sum test was used for continuous variables. A *p* value of <0.05 was considered significant. All statistical analyses were performed on JMP software (SAS, Cary, NC).

## Results

### MM Cohort

**Fig. 1:**
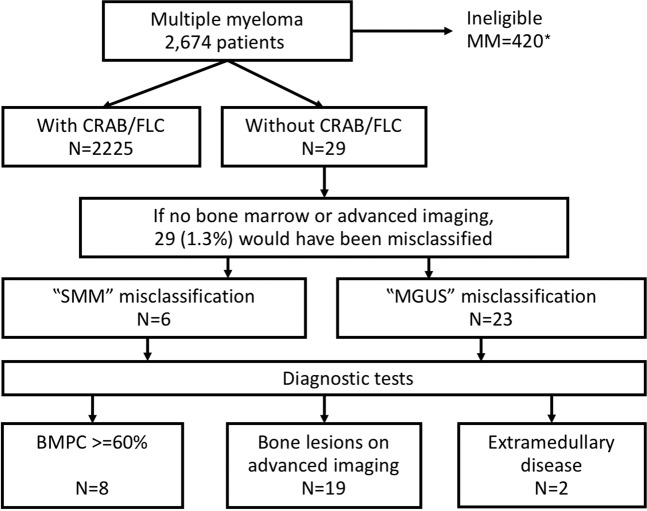
Myeloma cohort included in the study. Patients without CRAB/FLC had laboratory values within reference range for calcium, creatinine, hemoglobin, and absence of lytic lesions assessed by conventional skeletal survey and an FLC ratio of <100. Those “with CRAB/FLC” had an abnormality detected in at least one of these variables. CRAB hypercalcemia, renal failure, anemia, bone lesions, FLC free light chains, SMM smoldering multiple myeloma, MGUS monoclonal gammopathy of undetermined significance, BMPC bone marrow plasma cells. *patients’ missing data for SMM risk stratification.

Of 2254 MM patients meeting the study inclusion criteria, BM findings and/or advanced imaging findings were independent factors in diagnostic classification of 29 (1.3% of) MM patients. Of these 29 MM patients without CRAB/FLC, 6 would have been labeled SMM and 23 labeled as MGUS had no advanced imaging or BM been done, Fig. 1. With BM and advanced imaging, the following was found: BMPCs ≥ 60% in 8 patients; bone lesions identified on advanced imaging in 19 patients (lytic lesions in 16, pathological vertebral compression fractures in 2 patients, and sclerotic lesions in 1 patient); and extramedullary disease in 2 patients (liver and prostate involvement, respectively).

Baseline characteristics of patients presenting without CRAB/FLC and with CRAB/FLC features are listed in Table 1. Median age was similar for the two groups, with a lower proportion of males seen in the without CRAB/FLC cohort. Markers of tumor burden like M-protein involved serum FLC, involved-to-uninvolved FLC ratio, beta-2 microglobulin, and BM plasmacytosis were all lower in the without CRAB/FLC cohort.

**Table 1:** Multiple myeloma cohort subcategorized (*n* = 2254).

Variable	Without CRAB/FLC^a^ (*n* = 29)	With CRAB/FLC^b^ (*n* = 2225)	*p* value
Age, median (range)	61 (40–79)	63 (22–95)	0.29
Male, %	45	60	0.12
Monoclonal protein, *n* (%)			0.9
IgG	17 (59)	1126 (52)	
IgA	6 (21)	467 (22)	
Light chain	5 (17)	481 (23)	
Other	1 (3)	73 (3)	
Serum M-spike, g/dL, median (IQR)	0.8 (0–1.8)	2.1 (0.4–3.6)	0.0009
M-spike category, *n* (%)			0.0005
≤1 g/dL	16 (57)	664 (33)	
1 < g/dL ≤ 2	8 (29)	312 (16)	
>2 g/dL	4 (14)	1026 (51)	
Missing	1	223	
Free light chain type			0.86
Kappa	17 (59)	1283 (58)	
Lambda	11 (38)	797 (36)	
Nil	1 (3)	128 (6)	
Involved sFLC level, median (IQR), g/dL	13.5 (3.5–47.8)	53.7 (7.3–215.5)	0.002
sFLC ratio, median (IQR)	17.9 (3.7–71.0)	72.7 (10.9–404)	0.008
≥100, *n* (%)	5 (21)	746 (45)	0.02
Urine M-spike, mg/24 h, median (IQR)	25 (0–167)	120 (0–735)	0.08
Albumin, g/dL, median (IQR)	3.6 (3.2–3.8)	3.5 (3.1–3.8)	0.3
Beta-2 microglobulin, μg/mL, median (IQR)	2.2 (1.8–3.2)	3.8 (2.6–6.5)	<0.0001
BMPCs, median % (IQR)	20 (14–60)	50 (27–70)	0.0003
BMPCs category, *n* (%)			0.0006
<10%	3 (10)	96 (4)	
10–20%	12 (41)	345 (16)	
21–59%	6 (21)	825 (38)	
≥60%	8 (28)	916 (42)	
Missing	0	43	
ISS			0.007
I	12 (55)	538 (27)	
II	8 (36)	825 (41)	
III	2 (9)	652 (32)	
Missing	8	210	
Cytogenetic abnormality, *n* (%)			
17p	1 (5)	148 (13)	0.71
4;14	1 (7)	106 (10)	>0.99
14;16	1 (7)	53 (5)	0.54

*CRAB* hypercalcemia, renal failure, anemia, bone lesions, *sFLC* serum-free light chain, *BMPCs* bone marrow plasma cells, *ISS* International Staging System.

^a^Without CRAB/FLC: calcium, creatinine, hemoglobin in reference range, no lytic lesions assessed by conventional skeletal survey, and FLC ratio < 100.

^b^With CRAB/FLC: had an abnormality detected in at least one of these variables.

Had no BMB or advanced imaging been done in the 29 MGUS-like MM patients, they would have been classified as low-risk MGUS (*n* = 0); low-intermediate-risk MGUS (*n* = 11); intermediate risk (*n* = 12); high-risk MGUS *(n* = 1); and unclassifiable due to missing FLC values (*n* = 5).

### Smoldering myeloma cohort

**Fig. 2:**
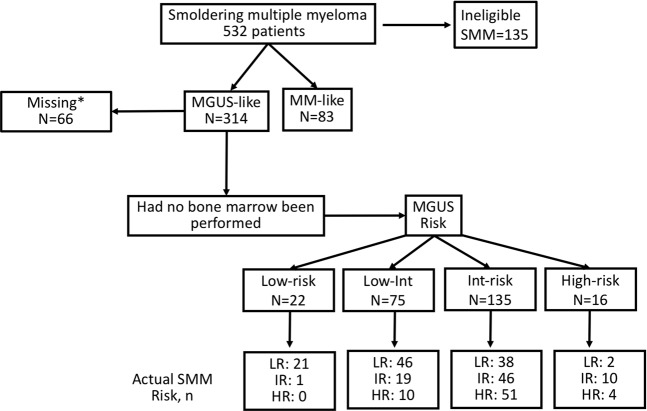
Smoldering multiple myeloma cohort included in the study. Those SMM patients with a serum M-protein of ≥3 g/dL or urinary M-protein ≥ 500 mg per 24 h were classified as “myeloma-like” with the remaining patients classified as “MGUS-like.” MM multiple myeloma, SMM smoldering multiple myeloma, MGUS monoclonal gammopathy of undetermined significance, Int intermediate, LR low risk, IR intermediate risk, HR high risk. An asterisk (*) represents patients’ missing data for SMM and MGUS risk stratification.

Of 397 SMM patients meeting study inclusion criteria, 314 (79%) would have been classified as MGUS without the BM (MGUS-like SMM), Fig. 2. The remainder were classified as MM-like SMM (*n* = 83) based on the size of the serum (< or ≥3 g/dL) and urine M-spike (< or 500 mg/24 h).

Demographic variables as well as monoclonal protein isotype and light chain subtype were similar for the two cohorts (Table 2). In addition to having higher M-spikes, serum FLCs, and BM infiltration, the MM-like SMM patients had higher creatinine and serum albumin, lower calcium. Consequently, a greater proportion of patients in the MM-like SMM cohort had high-risk SMM (74% for MM-like SMM vs 27% for MGUS-like SMM, *p* < 0.0001).

**Table 2:** Smoldering myeloma cohort subcategorized (*n* = 397).

	MGUS-like^a^ (*n* = 314)	Myeloma-like^b^ (83)	*p* value
Age, median (range)	66 (30–92)	65 (30–90)	0.63
Male, %	61	64	0.61
Monoclonal protein, *n* (%)			0.14
IgG	221 (71)	67 (82)	
IgA	57 (18)	7 (8)	
Light chain	17 (5)	5 (6)	
Other	18 (6)	3 (4)	
Light chain type			0.19
Kappa	187 (60)	58 (71)	
Lambda	121 (39)	23 (28)	
Nil	4 (1)	1 (1)	
Hemoglobin, g/dL, median (IQR)	12.5 (11.1–13.6)	12.0 (11.0–13.5)	0.16
Low hemoglobin, *n* (%)	73 (23)	20 (24)	0.88
Creatinine, mg/dL, median (IQR)	1 (0.9–1.2)	1.1 (0.9–1.4)	0.003
Elevated creatinine, *n* (%)	51 (16)	24 (29)	0.01
Calcium, mg/dL, median (IQR)	9.4 (9.1–9.8)	9.2 (8.9–9.7)	0.03
Elevated calcium, *n* (%)	27 (8)	7 (8)	>0.99
Albumin, g/dL, median (IQR)	3.5 (3.3–3.8)	3.7 (3.3–3.9)	0.0009
SFLC			
Involved SFLC level, median (IQR), g/dL	7.9 (2.9–22.7)	23.9 (4.5–58.2)	0.002
SFLC ratio, median (IQR)	6.9 (2.4–28.7)	33.5 (6.9–90.3)	<0.0001
SFLC ratio > 20, *n* (%)	78 (32)	31 (60)	0.0002
Missing	68	31	
Serum M-spike, g/dL, median (IQR)	1.6 (0.8–2.2)	3.3 (2.8–3.7)	–
M-spike category, *n* (%)			
≤1 g/dL	92 (29)	13 (16)	
1 < g/dL ≤ 2	122 (39)	4 (5)	
>2 g/dL	100 (32)	65 (79)	
Urine M-spike, mg/24 h, median (IQR)	0 (0–80)	150 (0–710)	
BMPCs, median % (IQR)	16 (11–25)	25 (13–30)	0.003
BMPCs category			<0.0001
<10%	–	12 (17)	
10–20%	215 (69)	16 (22)	
>20%	95 (31)	44 (61)	
Missing	4	11	
Mayo risk stratification SMM, *n*	248	46	<0.0001
Low, %	43	4	
Intermediate, %	31	22	
High, %	26	74	
Mayo risk stratification for MGUS, *n*	248	52	0.005
Low, %	9	2	
Low-intermediate, %	30	11	
Intermediate, %	55	79	
High, %	6	8	
Cytogenetic abnormality, *n* (%); missing			
17p	6 (4); 177	3 (12); 59	0.13
t(4;14)	7 (6); 188	4 (16); 58	0.09
t(14;16)	3 (2); 187	0; 58	>0.99

*MGUS* monoclonal gammopathy of undetermined significance, *sFLC* serum-free light chain, *BMPCs* bone marrow plasma cells, *SMM* smoldering multiple myeloma, *IQR* interquartile range.

^a^Myeloma-like: serum M-protein of ≥3 g/dL or urinary M-protein ≥ 500 mg per 24 h.

^b^MGUS-like: serum M-protein of <3 g/dL and urinary M-protein < 500 mg per 24 h.

Had no BM been performed, these MGUS-like SMM would have been incorrectly called MGUS, with 97 of them being classified as low- or low-intermediate-risk MGUS and 151 Intermediate- or high-risk MGUS; 66 had missing information precluding classification. Amongst patients with data available of MGUS risk classification 64% (158/248) had an abnormality in hemoglobin, calcium, renal function, albeit insufficient to classify them as active MM. Of the 90 (36%) patients without an abnormality in these laboratory parameters, MGUS risk stratification would have been 3% (*n* = 3) low, 29% (26) low-intermediate, 63% (*n* = 57) high-intermediate, and 4% (*n* = 4) high risk. Of the 29 MGUS-like patients without a laboratory abnormality who would have been classified as low or low-intermediate-risk MGUS, only four (1% of whole SMM cohort) had high-risk SMM features. Among the MGUS-like SMM, none of those who would have been classified as low-risk MGUS and only 13%(10/75) of the low-intermediate-risk MGUS had high-risk SMM (Fig. 2).

### Comparison to MGUS patients

There were 5863 MGUS patients in the study, 17% (*n* = 1024) of whom had a BM performed. As shown in Supplementary Table 1, the MGUS patients who had a BM performed were younger and had lower hemoglobin values, slightly higher calcium and creatinine values, higher FLC, and higher serum M-spike levels corresponding to higher MGUS and SMM risk stratification categories than those who did not have BM performed. Given the fact that the MGUS patients who had the BM performed were “worse” in all measures, these 1024 MGUS individuals were used as a comparator group to the MGUS-like MM and MGUS-like SMM cohorts, Table 3. These better characterized MGUS patients were predominantly low- or low-intermediate risk (75%); only 3.1% of them were intermediate risk, and none were high-risk MGUS. As would be expected, patients with MGUS-like MM and MGUS-like SMM had higher serum M-spike, free light chain levels, and BM plasma cells than did the MGUS patients. Surprisingly, the MGUS patients had lower serum albumin and hemoglobin and higher serum creatinine values than their MGUS-like MM and SMM counterparts.

**Table 3:** Comparison of baseline features among patients with plasma cell dyscrasias who would be considered MGUS if no baseline bone marrow or advanced imaging.

Variable	MM without CRAB/FLC (*n* = 29)	MGUS-like SMM (*n* = 314)	MGUS with BM (*n* = 1024)	*p* value
Age, median (IQR)	61 (54–69)	66 (57–72)	66 (57–74)	0.12
Male, %	45	61	61	0.21
Isotype: IgG/IgA/LC/other, %	59/21/21/0	71/19/5/6	66/10/7/17	<0.0001
Serum M-spike, g/dL, median (IQR)	0.8 (0–1.8)	1.6 (0.9–2.2)	0.4 (0–0.9)	<0.0001
M-spike category, *n* (%)				<0.0001
≤1 g/dL	16 (57)	92 (29)	767 (79)	
1 < g/dL ≤ 2	8 (29)	122 (39)	170 (18)	
>2 g/dL	4 (14)	100 (32)	30 (3)	
Missing	1	0	57	
Light chain type				0.82
Kappa	17 (59)	187 (60)	587 (59)	
Lambda	11 (38)	121 (39)	402 (40)	
Nil	1 (3)	4 (1)	14 (1)	
Involved sFLC level, median (IQR), g/dL	13.5 (3.5–47.8)	7.9 (2.9–22.7)	2.9 (1.8–6.0)	<0.0001
sFLC ratio, median (IQR)	17.9 (3.7–71.0)	6.9 (2.4–28.7)	1.7 (1.1–3.6)	<0.0001
SFLC ratio > 20, *n* (%)	12 (52)	78 (32)	41 (7)	<0.0001
Missing	4	67	444	
BMPCs, median % (IQR)	20 (13–60)	16 (11–25)	3 (2–5)	–
Albumin, g/dL, median (IQR)	3.6 (3.2–3.8)	3.5 (3.2–3.7)	3.4 (3.0–3.7)	0.0002
Beta-2 microglobulin, μg/mL, median (IQR)	2.2 (1.8–3.2)	2.6 (2.0–3.5)	2.6 (1.9–4.0)	0.2
Hemoglobin, g/dL, median (IQR)	13.7 (12.8–14.5)	12.5 (11.2–13.6)	12.4 (10.6–13.8)	0.0004
Creatinine, mg/dL, median (IQR)	0.8 (0.7–1.0)	1 (0.9–1.2)	1.1 (0.9–1.3)	<0.0001
Calcium, mg/dL, median (IQR)	9.4 (9.1–9.6)	9.4 (9.1–9.8)	9.5 (9.1–9.8)	0.6
Mayo risk stratification for SMM, *n*	23	248	552	
Low, %	39	43	90	
Intermediate, %	17	31	9	
High, %	44	26	1	
Mayo risk stratification for SMM (sans BM), *n*	23	248	552	<0.0001
M-spike ≤ 2 and FLC ≤ 20, %	43	51	90	
M-spike ≤ 2 or FLC ≤ 20, %	43	36	9	
M-spike > 2 and FLC > 20, %	14	13	1	
Mayo risk stratification for MGUS, *n*	24	248	563	<0.0001
Low, %	0	9	38	
Low-intermediate %	46	30	37	
Intermediate %	50	55	23	
High %	4	6	2	

*MGUS* monoclonal gammopathy of undetermined significance, *sFLC* serum-free light chain, *BMPCs* bone marrow plasma cells, *SMM* smoldering multiple myeloma, *IQR* interquartile range, *BM* bone marrow, *w*/*o* without.

### Overall survival

With a median follow-up of 30.1 months (IQR 5.8, 81.6 months) of the 1553 patients, the 5-year overall survival estimates for the MGUS-like MM, MGUS-like SMM, and MGUS with BM performed were 62.5, 71.9, and 67.1%, *p* = 0.33.

## Discussion

Assessment of the BM plays a key role in differentiating patients presenting with MGUS from SMM and MM and for prognostication in all groups^5–8^. The clinical presentations of patients with SMM and MM are variable, with clinical and laboratory signs guiding physicians on the need for more invasive investigation with BM or advanced imaging. However, the prevalence of MGUS increases with age, seen in 8.7% of patients above the age of 80. At presentation, nearly three-quarters of MGUS patients are low-risk or low-intermediate risk, and with increasingly sensitive techniques for screening for MGUS, that proportion will increase. It was our hypothesis (and a part of consensus guidelines^13^) that performing a BM and advanced imaging in all patients is unnecessary.

We demonstrate that only 0.3% of patients with MM present without myeloma defining events other than excessive BM plasmacytosis. The vast majority (98.7%) of MM patients presented with some objective abnormality in CRAB features or with a markedly elevated ratio of involved-to-uninvolved FLC. More often, CRAB type abnormalities are the initial triggers for physicians to identify a monoclonal protein. Among such patients a BM with genetic risk assessment is mandatory to prognosticate the MM.

Amongst the SMM population we found that the BM was the defining feature in upstaging a diagnosis of MGUS to SMM in 79% of patients, 72% of whom had low- or intermediate-risk SMM. In addition, further analysis of this group revealed that in 99% there were other potential triggers for further investigation including laboratory parameters not quite meeting myeloma defining event thresholds, including reduced hemoglobin or renal function; increased calcium; or greater low-risk MGUS. This means that omitting a BM in a patient without laboratory abnormalities in hemoglobin, calcium, and renal function and who has low-risk MGUS will result in missed diagnosis of MM or SMM in <1% of patients. If the criteria are broadened to omit BM in patients who appear to have low-intermediate-risk MGUS, 13% of high-risk SMM patients would be understaged as MGUS as would less than 0.5% of MM patients. If we take into account abnormalities in hemoglobin, calcium, and renal function as triggers for BM, then the number of high-risk SMM patients being understaged as MGUS is even lower (*n* = 4, 1% of SMM cohort). This translates into the potential of avoiding a BM more than 40% of our MGUS patients who had a BM performed—a percentage that would be greatly expanded if we included those MGUS patients in our series who did not have a BM performed. Missing a diagnosis of high-risk SMM myeloma (or MM without CRAB) at first presentation is not catastrophic even in the setting of emerging data demonstrating better progression-free and overall survival among high-risk SMM patients receiving lenalidomide-based therapy^14,15^ since these patients will be serially followed with blood work. Such an approach has the potential to avoid unnecessary investigations that may be associated with clinical risk as well as financial toxicity, which is especially relevant in the elderly. Our approach is further supported by the fact that the overall rates of the MGUS-like MM, the MGUS-like SMM, and the better characterized MGUS patients were similar, with the best overall survival in the MGUS-like SMM patients.

Our data support current recommendations stating that patients with low-risk uncomplicated MGUS (IgG MGUS, M-spike < 1.5 g/dL and normal free light chain ratio) or light chain MGUS (SFLC ratio < 8) do not require investigation with a BM^13^. Our data also give credence to expanding this recommendation one step further to suggest that low-intermediate MGUS patients also may not require a BM.

There are limitations to our study. It is retrospective with inherent biases and conducted over a period of 16 years. The diagnostic criteria for MM have changed over the study period, although we accounted for this by reclassification of patients according to the updated criteria. Nevertheless, this has also led to a significant change in approach to investigating these patients over the study period. For example, advanced imaging was used inconsistently in our cohort. Presence or absence of lytic lesions for the majority of patients during the study was based on skeletal survey with plain radiographs. In addition, the serum FLC assay, a vital component in diagnosing and risk stratifying plasma cell disorders, was introduced into routine hematology practice in 2002, with a slower adoption rate by other practitioners. A majority of the MGUS patients had data missing on this variable in our study as did a fraction of our MM and SMM patients.

Despite these limitations our study provides data supporting the safe deferral of invasive investigation with BM in patients with low-risk and low-intermediate MGUS that do not have concerning features on history, physical examination, or routine laboratory tests.

## Supplementary information

Reporting Checklist

Supplementary Table 1
