# Supplementary material for: The role of bone marrow biopsy in patients with plasma cell disorders: should all patients with a monoclonal protein be biopsied?
Source: Blood Cancer J. 2020 May 6;10(5):52. doi: 10.1038/s41408-020-0319-0 (PMC7203099; doi:10.1038/s41408-020-0319-0)
Supplement: Supplementary file 2 — Supplementary Table 1 [file 41408_2020_319_MOESM2_ESM.docx]

**Supplementary Table 1. Comparison of MGUS patients with and without bone marrow assessment.**

| **Variable** | **MGUS w/o BM n=4839** | **MGUS with BM n=1024** | **P value** |
| --- | --- | --- | --- |
| Age, median (IQR) | 69 (60-77) | 66 (57-74) | <0.0001 |
| Male, % | 59 | 61 | 0.19 |
| Monoclonal Protein, n (%) |  |  | <0.0001 |
| IgG | 2967 (62) | 668 (66) |  |
| IgA | 554 (11) | 99 (10) |  |
| Light Chain | 174 (4) | 66 (7) |  |
| Other | 1091 (23) | 176 (17) |  |
| Serum M-spike, g/dL, median (IQR) | 0 (0-0.5) | 0.4 (0-0.9) | <0.0001 |
| M-spike category, n (%) |  |  | <0.0001 |
| ≤1g/dL | 4206 (91) | 767 (79) |  |
| 1< g/dL ≤2 | 358 (8) | 170 (18) |  |
| >2g/dL | 41 (1) | 30 (3) |  |
| Missing | 234 | 57 |  |
| Light Chain type |  |  | 0.69 |
| Kappa | 2719 (57) | 587 (59) |  |
| Lambda | 1974 (42) | 402 (40) |  |
| Nil | 61 (1) | 14 (1) |  |
| Involved sFLC level, median (IQR), g/dL | 2.7 (1.7-5.0) | 2.9 (1.8-6.0) | 0.02 |
| sFLC ratio, median (IQR) | 1.4 (1-2.4) | 1.7 (1.1-3.6) | <0.0001 |
| SFLC ratio >20, n (%) | 30 (3) | 41 (7) | 0.0002 |
| Missing | 3832 | 449 |  |
| Albumin, g/dL, median (IQR) | 3.4 (3.1-3.7) | 3.4 (3.0-3.7) | 0.009 |
| Beta 2 Microglobulin | 2.9 (2.0-4.7) | 2.6 (1.9-4.0) | 0.002 |
| Hemoglobin, g/dL, median (IQR) | 13.0 (11.6-14.2) | 12.4 (10.6-13.8) | <0.0001 |
| Creatinine, mg/dL, median (IQR) | 1.1 (0.9-1.4) | 1.1 (0.9-1.3) | 0.0075 |
| Calcium, mg/dL, median (IQR) | 9.5 (9.2-9.8) | 9.5 (9.1-9.8) | 0.003 |
| Mayo Risk Stratification for SMM (sans BM), n (%) |  |  | <0.0001 |
| M-spike <= 2 and FLC <= 20 | 945 (96) | 497 (90) |  |
| M-spike <= 2 or FLC <= 20 | 36 (4) | 50 (9) |  |
| M-spike > 2 and FLC > 20 | 1 (<1) | 5 (1) |  |
| Mayo Risk Stratification for MGUS, n (%) |  |  | 0.0005 |
| 0 | 416 (42) | 214 (38) |  |
| 1 | 420 (42) | 211 (37) |  |
| 2 | 143 (14) | 127 (23) |  |
| 3 | 14 (2) | 11 (2) |  |
| missing | 3846 | 461 |  |
